# Letter to the editor: Comments on “Mitigating potential public health risks and challenges from hazardous materials contained in electronic waste items in a developing country setting”

**DOI:** 10.5620/eaht.2023021

**Published:** 2023-10-27

**Authors:** Slamet Wardoyo

**Affiliations:** Department of Environmental health, Poltekkes Kemenkes Surabaya, Surabaya, Indonesia

Dear Editor,

I am writing to express my opinion on the article “Mitigating potential public health risks and challenges from hazardous materials contained in electronic waste items in a developing country setting”. Electronic wastes, which contain hazardous chemicals, are rapidly generated in poor countries due to demand for affordable near-end-of-life internetenabled gadgets that soon wear out and are improperly disposed due to ignorance, throw-away mentality and dearth of waste management infrastructure” by O.C. Eneh et al. published in Environmental Analysis Health and Toxicology 2023; 38 (1): e2023001. The article provides a comprehensive overview of the environmental and health impacts of electronic waste (e-waste) containing hazardous chemicals such as mercury, polychlorinated biphenyls (PCBs), cadmium, lead and beryllium oxide.[1] In addition, the article makes some recommendations for policy changes and mitigation strategies to deal with the issue of electronic waste in developing countries. I want to express my gratitude to the authors for their efforts to bring attention to this critical matter and to suggest potential solutions that could help achieve the Sustainable Development Goals (SDGs) targets that are related to the management of hazardous chemicals and waste.

Having said that, I do have a few concerns as well as some suggestions with regard to the article. To begin, I believe that the article missed an opportunity to be more objective and balanced if it had acknowledged the positives and opportunities associated with the recycling and reuse of e-waste, particularly for developing nations. E-waste consists of valuable metals and materials that can be extracted and repurposed for a variety of reasons, including the production of energy, the manufacturing of goods, and the development of new ideas. Recycling and reusing electronic waste can also provide marginalized groups with opportunities for gainful employment, increased income, and increased social inclusion. Some examples of these groups include informal waste collectors and processors. For this reason, I believe that the article should have discussed the potential of e-waste as a resource, rather than focusing solely on the unfavorable aspects of this topic.

Second, I believe that the article missed an opportunity to be more specific and evidence-based by not supplying sufficient amounts of data and examples to back up the claims and recommendations. For instance, the article makes a passing reference to the rapid production of electronic waste in developing nations, but it does not provide any statistics or sources to quantify the scope of the issue or the patterns that it exhibits. The article also suggests the formulation of appropriate environmental health education technology policy (AEHETP) to guide stakeholders in designing education, preventive, therapeutic and decontamination plans, but does not explain what such a policy would entail or how it would be implemented and evaluated.[1] The article might have been able to persuade the reader more and provide more useful information if it had included additional information and references that were more concrete and pertinent.

Third, I believe that the article could have been more interesting and comprehensible if it had been written in a language and format that were more user-friendly. The article is written in a very academic and technical style, which may put off some readers or cause them to become confused if they are not already familiar with the subject matter or the terminology. In addition, there are no visual aids, such as tables, figures, or graphs, included in the article to illustrate the primary points and findings. The article might have been more interesting to read and easier to comprehend if it had been written in language that was less complicated and more transparent, and if it had included some visual elements to improve the way the message was presented and communicated.

In conclusion, I believe that the article makes an important contribution to the body of research on hazardous chemicals and electronic waste; however, I believe that it could be improved by addressing the concerns and suggestions that were presented earlier. I have high hopes that the authors will give some thought to revising and updating their article in order to make it more well-rounded, evidence-based, specific, interesting, and approachable for the audience.

## References

[1] Onyenekenwa C, Eneh OC, Eneh CA, Eneonwo CI, Okosun A, Emenuga V, Obi NI (2023). Mitigating potential public health risks and challenges from hazardous materials contained in electronic waste items in a developing country setting. Environmental Analysis Health and Toxicology.

